# Executive Function, Working Memory, and Verbal Fluency in Relation to Non-Verbal Intelligence in Greek-Speaking School-Age Children with Developmental Language Disorder

**DOI:** 10.3390/brainsci11050604

**Published:** 2021-05-08

**Authors:** Asimina M. Ralli, Elisavet Chrysochoou, Petros Roussos, Kleopatra Diakogiorgi, Panagiota Dimitropoulou, Diamanto Filippatou

**Affiliations:** 1 Department of Psychology, National and Kapodistrian University of Athens, 15784 Athens, Greece; roussosp@psych.uoa.gr (P.R.); filipd@psych.uoa.gr (D.F.); 2 School of Psychology, Aristotle University of Thessaloniki, 54124 Thessaloniki, Greece; echrysoc@psy.auth.gr; 3 Department of Education & Social Work, University of Patras, 26504 Patras, Greece; kdiakogiorgi@upatras.gr; 4 Department of Psychology, University of Crete, 74100 Crete, Greece; p.dimitropoulou@uoc.gr

**Keywords:** Developmental Language Disorder, executive functions, working memory, verbal fluency, non-verbal intelligence, school-age children

## Abstract

Developmental Language Disorder (DLD) is often associated with impairments in working memory (WM), executive functions (EF), and verbal fluency. Moreover, increasing evidence shows poorer performance of children with DLD on non-verbal intelligence tests relative to their typically developing (TD) peers. Yet, the degree and generality of relevant difficulties remain unclear. The present study aimed at investigating WM capacity, key EFs and verbal fluency in relation to non-verbal intelligence in Greek-speaking school-age children with DLD, compared to TD peers (8–9 years). To our knowledge, the present study is the first to attempt a systematic relevant assessment with Greek-speaking school-age children, complementing previous studies mostly involving English-speaking participants. The results showed that children with DLD scored lower than TD peers on the non-verbal intelligence measure. Groups did not differ in the inhibition measures obtained (tapping resistance to either distractor or proactive interference), but children with DLD were outperformed by TD peers in the WM capacity, updating, monitoring (mixing cost), and verbal fluency (phonological and semantic) measures. The effects showed limited (in the case of backward digit recall) or no dependence on non-verbal intelligence. Findings are discussed in terms of their theoretical and practical implications as well as in relation to future lines of research.

## 1. Introduction

Developmental Language Disorder (DLD) (previously known as Specific Language Impairment or Language Impairment) in children has been the focus of scientific attention for the last three decades. However, the mechanisms underlying this disorder remain unclear. There is growing evidence that besides linguistic factors, domain-general cognitive abilities may contribute to the difficulties associated with DLD [1,2]. According to Tomas and Vissers [3], children with DLD seem to face multiple cognitive deficits which impede typical language acquisition and development. Fine-grained evaluations of cognitive abilities may thus contribute to accurate, early identification and diagnosis of DLD, as well as to a more comprehensive description of DLD profiles [4,5], that could, in turn, increase the effectiveness of relevant interventions, tailoring them to the linguistic as well as cognitive strengths and weaknesses of each child with DLD.

In such a context, the present study attempts a systematic exploration of cognitive capacities and functions in Greek-speaking school-age children with DLD. Specifically, we focus on working memory (WM) capacity, namely the ability to concurrently maintain and process information [6,7]). We also study key executive functions (EFs) in DLD, as defined by Miyake and colleagues [8]; that is, focusing on updating, set-shifting (otherwise, task switching), and inhibition. Such a tripartite structure has been confirmed in studies involving preschoolers [9] as well as school-age children [10]. Moreover, we aim for a comprehensive approach to the study of inhibition. In doing so, we adopt the perspective of Friedman and Miyake [11] and rely on their taxonomy of inhibition tasks, thus differentiating evidence with regard to prepotent response inhibition, resistance to distractor interference, and resistance to proactive interference. Moreover, given inconclusive evidence regarding verbal fluency in children with DLD—mostly stemming from studies with English-speaking children—we focus on two relevant indices; phonological and semantic fluency. Though the latter capacities have been related to language and executive control to different degrees, very few studies have discussed difficulties of children with DLD in both fluency aspects. The combined investigation of the aforementioned factors has scarcely been attempted, whereas there is no relevant evidence with Greek-speaking school-age children with DLD; the latter remains an under-examined population in the DLD literature. Finally, since more and more researchers emphasize the need to consider non-verbal intelligence as part of the DLD profile [4,12], the present study also investigates whether any differences observed between children with DLD and typically developing (TD) peers in WM, EFs and verbal fluency, are independent of non-verbal intelligence.

### 1.1. Developmental Language Disorder

Developmental Language Disorder is defined as difficulty with the acquisition of language skills (expressive and/or receptive) in the absence of any obvious cause, given normal non-verbal IQ and no primary physical disabilities, neurological disorder, or mental illness [13,14,15]. DLD can be reliably diagnosed after the age of 4 years [16,17,18] and is roughly characterized as a lag of about two years in the development of language skills [19]. It is observed in 5–10% of the population [20,21], which explains the significant research attention drawn to this disorder over the last 30 years [4,22,23,24].

Both genetic [1,25,26,27,28,29] and environmental risk factors (e.g., low socioeconomic status–SES—or fewer years of parental education) [27,30] have been identified for DLD. Moreover, both language-specific and domain-general accounts of the disorder have been proposed. Within the former, children with DLD are suggested to face difficulties in linguistic processing, mostly in grammatical processing, implying that other cognitive or neural processes remain largely intact as they develop [31,32,33]. Such research focuses on establishing clinical markers of DLD in the language domain, which could be used for screening and intervention. On the other hand, several researchers claim that children with DLD have additional domain-general cognitive processing impairments. Specifically, DLD is approached as multidimensional, encompassing deficits in both the domain of language acquisition [34,35] as well as co-morbid problems in cognitive domains, including those of attention [36], information processing [2], simultaneous processing [37], and verbal fluency [38,39,40,41]. For example, according to the *Procedural Deficit Hypothesis* (PDH), introduced by Ullman and Pierpont [42], DLD can be largely explained by abnormal development of brain structures that support procedural memory; this, in turn, is assumed to result to grammatical impairments as well as deficits, however, in the development and execution of motor and cognitive processes. And although some researchers have suggested a single impaired cognitive mechanism underlying DLD [43,44], relying on comparisons of children with and without DLD on capacities such as sustained attention [45,46,47] or auditory perception [48,49,50,51], others, have related the disorder to impairments in at least two cognitive processes [1], emphasizing the relation of linguistic and higher-order processing (e.g., EF) in children with DLD.

In summary, the underlying causes for DLD are still not well understood. Although the diagnosis of the disorder is based on impaired language acquisition in the absence of cognitive impairment, more and more researchers are questioning such exclusionary criteria, suggesting the need for clearer insight to be obtained into the interaction of higher-order cognitive functioning with language development in children with DLD [12,52,53].

### 1.2. Non-Verbal Intelligence in Children with DLD

Traditionally, the diagnosis of DLD has required non-verbal IQ within normal limits [15,54]. However, there are studies that have reported poorer non-verbal intellectual functioning in children with this disorder relative to TD peers [4,12,55]. For example, in a recent meta-analysis, Gallinat and Spaulding [12] found that children with DLD performed on average 10 points below the TD children on non-verbal intelligence [56]. The researchers actually suggest that poorer performance in non-verbal intelligence tests is part of the clinical profile of children with DLD. Moreover, in a recent project (CATALISE) which aimed for a consensus on terminology for unexplained language problems in children, it was overall claimed that discrepancy between verbal and non-verbal ability is not indicative of underlying etiology in DLD [13]. For example, identical twins with language problems often had very similar language profiles but differed in terms of non-verbal ability, with one twin meeting traditional discrepancy criteria for DLD and the other one not doing so [34]. Furthermore, for a child without an identified syndrome, the level of non-verbal IQ does not appear to determine responsiveness to therapy. Therefore, Bishop [57] claimed that there is no justification for using verbal vs. non-verbal ability discrepancy as a diagnostic criterion, as it might exclude from diagnosis, and thus intervention, children with DLD that could have otherwise benefited. It seems that the non-verbal IQ diagnostic criterion of DLD has been at the center of an ongoing debate within the field [58,59].

### 1.3. Working-Memory Capacity in Children with DLD

Working-memory capacity regards an individual’s ability to concurrently process and store information, keeping it active and accessible as long as the task at hand necessitates that. There seems to be a growing consensus that the crucial element of WM is controlled attention and coordination of information flow through WM (attributed to the central executive of the Baddeley and Hitch 1974 model [6,7] and tapped by complex span tasks), rather than its storage capacity [60]. Such executive attentional control in WM constitutes a powerful predictor of language development [61,62,63] and more generally, a strong determinant of learning capacity and scholastic attainment through the school years (regarding both literacy and numeracy) [64,65].

To no surprise, WM capacity has therefore received attention in the DLD literature as well. For example, besides performance in EF tasks (see discussion below), difficulties in WM (verbal and visuospatial; as tapped by cognitive measures as well as parental reports) differentiated preschool children with DLD and TD peers in the Vugs et al. study [66]. In a more recent study, Vugs and colleagues [67] showed varying associations of WM with the linguistic skills of children with DLD (receptive and expressive vocabulary, oral comprehension, and syntactic development). Moreover, limitations in WM capacity (as tapped by verbal tasks) have been found to constrain language development of school-age children with DLD beyond any intelligence effects [68,69,70] or general language ability effects (see also Archibald and Gathercole [71], where relevant visuospatial STM/WM effects were less marked or insignificant). In one of the few systematic explorations of cognitive functioning in children with DLD (8- to 14-year-olds), Henry et al. [68] reported smaller WM capacity, less efficient inhibition (prepotent response inhibition) and planning, and lower fluency among children with DLD, but no differences in task switching relative to TD peers. It is noted that the deficits observed were not constrained to verbal tasks and were independent of any IQ effects (verbal and non-verbal) [72]. Yet, such systematic explorations of cognitive functioning in children with DLD remain few. Given the relevant scope of the present study, the following section reviews evidence on key EFs in relation to DLD, focusing on updating in WM and task switching, and differentiating findings with regard to distinct inhibition functions; that is, response inhibition, resistance to distractor interference, and the least investigated in the DLD literature, resistance to proactive interference function [11].

### 1.4. Executive Functions in Children with DLD

#### 1.4.1. Updating in Children with DLD

Updating regards constant monitoring and rapid addition or deletion of WM contents in relation to the current task demands [73]. The specific function has been studied much less than WM capacity in relation to DLD. For example, in the aforementioned systematic investigation of EFs in children with DLD, Henry et al. [68] did not include updating measures. Kapa and Plante [74] did not discuss relevant findings in their review of EF difficulties of children with DLD, whereas only two studies (discussed below) are reported in the Montgomery et al. [75] discussion of memory and language capacities in children with DLD [39].

In one of the few relevant studies, Im-Bolter et al. [76] assessed updating along with switching and attentional capacities in school-age children (7- to 12-year-olds). Researchers observed greater difficulties in the DLD group relative to the TD children, but only in the one-back condition of the dot-pattern recognition n-back task used. Let us note, however, that this condition sets minimum updating demands, requiring the child to compare each incoming stimulus with the one previously viewed (pressing the “yes” button when it is the same) and discard stimuli more than one-back. Yet, both children with DLD and TD peers performed at chance levels on the two-back condition of the task (where the “yes” button should be pressed when the stimulus is the same as the one viewed two trials back). This pattern limited further exploration of updating in children with DLD, since the two-back task version (also used in the present study) is considered a more valid measure of the specific function relative to its one-back version, and has, thus, more widely been used in relevant investigations. Specifically, the two-back version is assumed to set demands on the executive control system for maintaining accurate representations of information that changes over time while monitoring the process and resisting proactive interference [77]. In a recent study, Evans et al. [78] did not observe significant differences between children with DLD and TD school-age peers (7- to 11-year-olds) in another updating measure employed (keep track task); neither were group differences observed on measures of attention switching and interference resolution. Null findings regarding updating were also obtained by Lukács et al. [39] in a study involving 8-year-old children with DLD and TD peers in verbal and non-verbal n-back tasks. Overall, evidence regarding updating in children with DLD remains scarce and inconclusive.

#### 1.4.2. Inhibition in Children with DLD

Inhibition encompasses a set of processes that are important for language development, as well as learning more generally. In their taxonomy, Friedman and Miyake [11] (see also Miyake and Friedman [73]) distinguish among three related functions: (a) prepotent response inhibition, which regards the deliberate overriding of dominant, automatic, or prepotent responses (e.g., tapped by stop-signal or Stroop tasks), (b) resistance to distractor interference, which regards the capacity to resolve conflict created by information that is irrelevant to the task at hand e.g., tapped by flanker tasks), and (c) resistance to proactive interference, that is, capacity to resist intrusions from information that was previously relevant but has since become irrelevant (e.g., tapped by tasks requiring recall of word lists drawn from the same category; see a relevant task in the methods section). Let us note that distractibility and inhibition scores have been found to correlate with standardized language measures in children with DLD, in line with the roles attributed to attention and inhibition in language processing more generally [39,78]. Overall, however, evidence regarding specific inhibition functions in children with DLD remains scarce and controversial.

Specifically, with regard to prepotent response inhibition, in one of the few systematic investigations of EFs in school-age children with DLD, Im-Bolter, Johnson, and Pascual-Leone [76] documented impairment in an antisaccade task. This is consistent with evidence regarding the preschool years: for example, Spaulding [79] found that preschoolers with DLD were less efficient in suppressing a prepotent, conflicting response in a stop-signal paradigm, whereas Roello et al. [80] reported similar findings in a study using a Stroop-like task (Day-Night). Yet, Noterdaeme et al. [81] found no differences between school-age children with DLD (elementary and high school) relative to TD children in a go/no go task. In line, differences were not observed in the Marton et al. [82] study, where the stop-signal task was used with 10- to14-year-old children with DLD (see, however, their finding regarding sensitivity to interference below). Nor were differences reported between children with DLD and TD children (mean age: 7.8 years) in the Lukács et al. [39] study, relying on accuracy and RT measures of Stroop task versions (verbal and non-verbal). Finally, in one of the few systematic explorations of EFs in children with DLD (8- to 14-year-olds), Henry et al. [68] found that children with DLD had difficulties in only one among the inhibition measures obtained (that is, in a non-verbal motor inhibition measure) in comparison to TD children.

With regard to a second inhibition function, Spaulding [79] reported decreased resistance to distractor interference, regardless of distractor modality (i.e., non-verbal auditory, linguistic, or visual), in preschoolers with DLD relative to TD peers. In line, Finneran et al. [46] found significant accuracy but not also speed differences in favour of TD preschoolers in a task tapping monitoring of the target stimulus and inhibition of distractors. In consistence, with regard to the elementary-school years, children with DLD were found more easily distracted by interfering items relative to TD peers [82], regardless of distractor type (e.g., words or pictures in the Victorino and Schwartz study [83] with 9- to 12-year-olds). Yet, Evans et al. [78] did not observe significant differences in interference inhibition between children with DLD and TD peers (7- to 11-year-olds).

Finally, we lack evidence on a third inhibition function in relation to DLD: that of resistance to proactive interference. As Marton et al. [82] noted, the difficulties observed in children with DLD in verbal complex span tasks (such as the ones employed in the present study) might actually reflect poor resistance to both distractor and proactive interference (i.e., inhibiting lexical items that had been the focus of previous searches). Accordigly, with this suggestion, elementary school-age children with DLD were found more susceptible to proactive interference than children in the control group, in the verbal experimental task used by Marton et al. [84].

#### 1.4.3. Task Switching in Children with DLD

Task switching regards the ability to shift flexibly between mental sets [8,73]. Relevant findings for children with DLD are not characterized by consistency [74]. Task switching difficulties have been observed in studies with preschoolers [37,80]. On the other hand, though Im-Bolter and colleagues [76] reported lower updating and attentional control-inhibition capacities in school-age children with DLD relative to TD peers, they found no differences between groups in task switching (see also Dibbets et al. [85]; Evans et al. [78]; Henry et al. [68] for similar findings with verbal and non-verbal switching measures). In their recent meta-analysis, however, Pauls and Archibald [86] did report poorer cognitive flexibility in children with DLD as tapped by switching tasks, the *Wisconsin Card Sorting Test*, as well as fluency tasks; however, this effect was smaller than the one regarding inhibition.

### 1.5. Verbal Fluency in Children with DLD

Verbal fluency tasks measure strategic search and retrieval of information stored in the mental lexicon and, thus, semantic memory [87]. They require participants to generate as many words as possible within one minute according to simple rules that target sounds (phonological fluency; e.g., items starting with particular letters, such as “f”, “a”, “s”) or semantic categories (semantic fluency; e.g., “animals” or “foods”) [88,89].

Verbal fluency tasks have been described as measures of EF [90,91], requiring goal-directed behaviors such as flexibility of thought, strategic planning, non-habitual responses, and error-monitoring, as well as tapping on search and retrieval of information from long-term memory. As noted above, for example, in their meta-analysis on EFs in DLD, Pauls and Archibald [86] viewed the verbal fluency tasks as reflective of a subtype of cognitive flexibility that is related to the notion of generativity or creative fluency [92]. On the other hand, verbal fluency has also been viewed as a language measure [93], indicating proficiency [94] as a function of vocabulary size [95,96]. Finally, there are researchers acknowledging the mixed demands set by verbal fluency tasks, thus, approaching them as *executive language tasks* [97]. Within this context, there have also been attempts to disentangle the demands set by phonological versus semantic fluency in relevant tests. For example, performance on semantic fluency tests has been suggested to largely depend on lexical-retrieval speed as well as on the employment of visualization strategies to support controlled retrieval; whereas performance on phonological fluency tests has been suggested to heavily rely on vocabulary knowledge (see discussion in Gordon et al. [98]).

Despite such links to both language and EF, there are surprisingly few studies regarding verbal fluency in children with DLD, with findings remaining inconclusive so far [39]. Weckerly et al. [41], for example, showed both semantic and phonological fluency difficulties in school-age children with DLD [38]. Rodríguez et al. [99] reported problems with verbal fluency in the expressive DLD group of their study (not also in the expressive-receptive group). Verbal fluency difficulties were also observed in the DLD groups of the Lukács et al. [39] and the Henry et al. [68] (coupled with difficulties in non-verbal fluency) studies with school-age children. On the other hand, there have been studies reporting insignificant semantic fluency differences between children with DLD and TD peers [100,101]. Besides, controversial findings remain limited in languages other than English. A recently published study in the language of the present sample (one of the very few in Greek; see Mengisidou et al. [102]) found poorer semantic fluency performance in school-age children with DLD relative to TD peers; yet, researchers have not also used a phonological fluency measure.

### 1.6. Rationale of the Present Study

According to the literature reviewed, although several studies have focused on the performance of children with DLD in different cognitive tasks, it is not easy to draw a clear overall picture of relevant strengths and weaknesses. Actually, very few studies have employed measures tapping both WM capacity and different EFs [37,39,68,76], whereas even fewer have involved populations other than English-speaking children. In the previous sections, we have attempted a review of findings regarding WM capacity (as tapped by complex span tasks) as well as key EFs [8] and verbal fluency in children with DLD. With regard to EFs, we have discussed evidence regarding updating, task switching, and inhibition, differentiating, however, among inhibition-related functions (response inhibition, resistance to distractor interference, and resistance to proactive interference) [11]. Let us note that these functions do not develop at the same rate through childhood (see discussion in Best and Miller [103,104]) and might not be equally vulnerable in children with developmental disorders. As Marton et al. [84] noted, for example, even if school-age children with DLD perform similarly to TD peers in response inhibition, as some studies have shown, this might not be the case with regard to resistance to interference, which develops later.

Within this context, the present study aimed at investigating WM capacity, key EFs, and verbal fluency in Greek-speaking children with DLD and TD peers in relation to non-verbal intelligence. Even though these cognitive capacities play a key role in learning and thus, school performance as well as daily life, relevant systematic assessments in children with DLD remain limited in number; whereas, to our knowledge, this is the first study attempting a comprehensive relevant investigation in Greek-speaking school-age children with DLD. More generally, few studies have so far focused on DLD populations speaking a language other than English, despite evidence on possible modulation of the language-cognition interplay by the characteristics of the developing language [63]. It is noted that our parallel interest in non-verbal intelligence stems from recent evidence [4,12,55,56], showing significant differences in non-verbal intelligence measures between groups of children with DLD and TD peers even if, by definition, DLD diagnosis implies typical non-verbal intelligence. As noted in the relevant section of the introduction, however, the non-verbal IQ diagnostic criterion of DLD is actually at the center of an ongoing debate [58,59]. Thus, non-verbal intelligence was assessed in our study not only to obtain insight into the intellectual functioning of participants and inform group formation (ensuring inclusion of participants with average intelligence in both groups), but also to consider its possible role in the demonstration of group differences in WM capacity, the different EFs studied, and verbal fluency [105].

We stated the following research questions:

1. Do Greek-speaking school-age children with DLD have lower scores than TD peers on non-verbal intelligence?
2. Are Greek-speaking school-age children with DLD outperformed by TD peers in tasks tapping (a) WM capacity, (b) key EFs; that is updating, the resistance to distractor interference and resistance to proactive interference functions of inhibition, and task switching, and (c) verbal (phonological or semantic) fluency (executive-language measures)?
3. Are any group differences in WM capacity, EFs, and verbal fluency independent of non-verbal intelligence?

We stated the following hypotheses:

We expected children with DLD to be outperformed by TD peers on the non-verbal intelligence measure (even if both groups are of average intelligence), based on increasing evidence showing such group differences. With regard to the second and third research questions, we opted for a general hypothesis, given inconclusive findings in the literature as well as scarce evidence with Greek-speaking school-age children with DLD. We expected differences in favor of the TD children in the cognitive measures obtained that would be independent of non-verbal intelligence.

## 2. Materials and Methods

### 2.1. Participants

Participants were 58 monolingual Greek-speaking children attending the third grade of elementary state schools in Athens: 29 children with DLD and 29 TD peers (mean age 8.6 and 8.9 years, respectively). All participants had average intelligence (with a standard score of 85 or above in the Raven’s Educational CPM/CVS (Raven [106]; standardized for use with Greek children by Sideridis et al. [107]). Children with a diagnosed hearing impairment, neurological disorder, ADD/ADHD, or autism spectrum disorder were excluded from the sample. We recruited children with DLD based on an existing relevant diagnosis from a speech and language pathologist, which was further confirmed based on our assessments. Specifically, children in this group were found to perform approximately 1.25 SDs or more below the mean on two standardized language measures, following Tomblin [108]. The language measures included the *vocabulary subtest* from WISC-III in Greek [109] and the *Sentence completion* subtest from the *Athena test for the Diagnosis of Learning Difficulties* [110]. All TD children performed within normal ranges in both standardized language tests.

The two groups were matched in terms of gender, χ^2^ (1, N = 58) = 0.624, *p* = 0.599 (DLD group: 15 boys, 14 girls; TD peers: 12 boys, 17 girls). Finally, given significant correlations between SES and cognitive development [111,112], a mean parental SES score was calculated for each participant, based on the sum of ratings regarding his/her mother’s and father’s (a) education level (from 0—did not finish elementary school, to 6—Master’s/PhD recipient), (b) type of occupation (0—unemployed, 1—blue collar, 2—white collar), and (c) position in occupation (0—unemployed to 6—big business owner or executive member of the private or public sector) [113,114]. The groups of children with DLD and TD peers were found matched on parental SES, *F*(1, 56) = 3.347, *p* = 0.073, η_p_^2^ = 0.056 (M_DLD_ = 6.05, M_TD_ = 7.10).

### 2.2. Design

This study stems from a wider research project examining reading and writing skills in Greek-speaking school-age children, in relation to their language skills and cognitive functions, as well as their motivation, academic emotions, and psychological needs. In the present investigation, we adopted a quasi-experimental design comparing children with and without DLD (matched on gender and parental SES) on non-verbal intelligence, WM capacity (as tapped by complex span tasks), different EFs (updating, task switching and the inhibition-related functions of resistance to distractor interference and resistance to proactive interference), and verbal fluency (phonological and semantic fluency). The relevant tasks were given in random order in the context of four half-hour assessment sessions (to avoid fatigue) conducted within the same month.

### 2.3. Tasks

#### 2.3.1. Screening Tools

##### Non-Verbal Intelligence

The Raven’s Coloured Progressive Matrices (CPM) test (Raven’s Educational CPM/CVS; Raven [106]; standardized for use with Greek children, Sideridis et al. [107]) was used to measure non-verbal intelligence (reasoning). Children are presented each time with a visual geometric design with a missing piece, and they are asked to pick among six choices the piece that should be filled in to complete the pattern. The test includes 36 items arranged in three sets. Items within a set become increasingly difficult, requiring greater capacity to encode and analyze information. A child’s score on the test is the sum of correct responses on all three sets.

##### Expressive Vocabulary

Participants were also given the vocabulary scale from the Wechsler Intelligence Scale for Children-WISC III, as standardized in Greek [109]. The test consists of 30 words and the child is asked to give an oral definition for each word presented (scored 0 to 2). The process is discontinued after four consecutive incorrect answers. A child’s score on the test is the sum of the scores received for all answers given until discontinuation/completion of the assessment.

##### Sentence Completion

Children were also assessed with the sentence completion subtest of the Athena test [110]. This test taps a child’s ability to exploit his/her experience with language as well as linguistic redundancies (mostly at the levels of grammar, semantics, as well as pragmatics) to fill in gaps in linguistic material. Specifically, children are orally presented with 32 simple sentences and they are asked to complete each one by producing the missing word or phrase (e.g., “Hives are the homes of…”, “He got angry, he got out of …”—“himself”, a metaphor-based Greek saying). A child’s total score is the sum of correct responses until completion of the assessment or its discontinuation after four consecutive incorrect responses.

#### 2.3.2. Main Assessment Tools

##### Working-Memory Capacity

Participants’ WM capacity was assessed with the backward digit recall and the listening recall tests of the Working Memory Test Battery for Children (Pickering and Gathercole [115], as adapted for use with Greek-speaking children by Chrysochoou [116]; see also [61,62,63,117]). These are two complex span tasks, requiring concurrent storage and processing of information; they were included in the sub-battery of the WMTB-C, assessing the central executive component of the Baddeley and Hitch [6,7] WM model. Besides their wide use in studies with TD children, such complex span tasks have been used in several studies involving children with DLD [39,68].

Specifically, in the backward digit recall test, children were asked to listen to a list of digits presented each time with a rate of one digit per second and recall it in reverse order. The test consists of a maximum of six blocks, each containing six lists of the same difficulty level (e.g., in the first block, the child is presented with lists of two digits, in the second block, with lists of three digits, etc.).

In the listening recall task, children listen to a set of short sentences (with the spoken duration of each kept up to 2 s). They are first asked to judge the veracity of each sentence in a set (e.g., “balls are square”—false, “scissors cut paper”—true) and then, to recall the final word of each sentence in the order it was presented (square, paper in the specific example). The test consists of a maximum of six blocks, including sets of increasing length (from one to six sentences per set). In both WM tests, testing ceases when children fail any three trials in a particular block. A child’s score on each test is the sum of his/her correct responses.

##### Updating

A 2-back version of the n-back task [118,119,120] was used to assess updating (see relevant assessments in studies with children with DLD [76]. Children are presented with a series of digits on the computer screen, and they are asked to press a specific key (j) on the keyboard when the target digit matches the one presented two trials earlier. Each digit is presented in black font on a white background for 500 ms, and it is followed by a blank screen for 2500 ms. Order of presentation was pseudorandomized with the same digit, however, not presented twice in a row. The task starts with a practice block of 22 digits, including two dummy trials in the beginning (which were not analyzed further) as well as seven targets. The experimental block consists of 62 digits, among which the first two are dummy trials, and 20 are targets. The number of targets recognized is suggested to reflect updating capacity. A measure of response speed (reaction time—RT—in ms) for the correctly recognized targets is additionally obtained.

##### Inhibition: Resistance to Distractor Interference

To measure the ability to resist interference from distractors, namely from information in the environment that is not relevant to the task at hand (see Friedman and Miyake [11]), we used the Eriksen Flanker task [121], as adapted for use with school-age children for the wider project that this study stems from (see design; [122]; the task was also used in [123]). Flanker-task versions have been used widely with TD children [114,124,125,126,127] as well as children with disorders or learning disabilities [128,129,130]. The task was developed and administered in E-Prime 2.0 software [131].

In each trial, children are presented with a fixation cross followed by the presentation of the target letter, which is either A or Λ (specifically, the corresponding Greek letters A and L). Participants are asked to identify this target letter; a left or a right mouse click corresponds to the letters A and L, respectively. In the neutral condition trials, the target is flanked by asterisks (i.e., **A** or **L**), whereas in the compatible and the incompatible condition trials, the target letter is flanked by the same or the alternative letter, respectively, in uppercase or lowercase (i.e., AAAAA, ααAαα, ΛΛΛΛΛ, λλΛλλ in the compatible condition; ΛΛAΛΛ, λλAλλ, AAΛAA, λλΛλλ). Though the target is always presented in uppercase, the flanker-letter case is differentiated so as to avoid any effects of superficial, low-level (perceptual), target-flanker letter similarity effects [132,133]. Stimuli are presented in black font on white background. In each trial, the duration of fixation varied randomly across trials from 0 to 1200 ms. The target-flanker/asterisk arrays remain on screen until response [129,130].

Participants first complete a practice block of 24 trials with feedback (i.e., information was provided on whether the response was correct or not, along with the relevant RT in ms). The main test phase consists of three blocks of 24 trials each with no feedback provided. Within each block, trial order is pseudo-randomized, so that the trial type (neutral, compatible, incompatible) is not identical on more than two consecutive trials.

The interference control measure used in the analyses of the present study is calculated based on the difference between participants’ mean RT on the incompatible and the compatible condition trials. Incompatible trials are expected to elicit slower responses relative to compatible ones, due to competing action schemas.

##### Inhibition: Resistance to Proactive Interference

We adopted the task developed by Borella et al. [134] to measure children’s capacity to resist proactive interference (a second core inhibition-related function according to Friedman and Miyake [11]). Specifically, in the practice block as well as in each of the four assessment task blocks, children are presented with three lists of words belonging to the same semantic category. Specifically, color words are used in the practice block and words referring to animals, fruits, professions, or body parts are included in the lists of the four assessment blocks. Word lists were translated from Italian to Greek and, as with the original task version, we placed emphasis on keeping the number of syllables in the words used similar across assessment lists in order to maintain memory demands equivalent.

Within each block, the words of each list are presented to the child on a computer screen with the use of PowerPoint presentation and at a rate of one word per 2.5 s. Children are asked to read each word in a list silently. They are then presented with a number on screen (different for each list) and are asked to count backward by two for 30 s (rehearsal prevention phase). A blank slide follows with a green tick symbol, and participants are required to produce orally as many words as they can recall from the list, regardless of presentation order. Block order was counterbalanced across participants.

Summing the number of correct responses for each list (first, second, and third) across the four (semantic category) blocks resulted in the calculation of two susceptibility to proactive interference indices per participant [134]. In each case, recall in list 1 was considered as a baseline in the assessment of the proactive interference build-up (given possible intrusions in lists 2 and 3 only). Susceptibility to PI for list 2 and list 3 was estimated using the formula (list 1–list 2) and (list 1–list 3). The average of the two was entered in the analyses as the overall susceptibility to proactive interference index. The smaller the average, the better the capacity to resist interference from information (words) that was relevant at some point, but should have been cleared afterwards so as to achieve task goals.

##### Task Switching

Participants’ ability to switch flexibly between tasks was assessed with the “How many—What number task” [135] (see Im-Bolter et al. [76] for use of the same task with children with DLD). Participants are presented with four types of stimuli cards consisting of either one (1 or 3) or three digits (1 1 1, 3 3 3). Two pure, non-switch blocks (each consisting of 4 practice and 24 assessment trials) are presented in the beginning in counterbalanced order across participants. Each pure block requires the application of one of two rules: in one block, participants are asked to identify the numeric value of the digits presented each time on the screen, pressing either 1 or 3 on the keyboard (What number block), whereas in the other pure block, they are asked to identify the number of digits presented on the screen using the same response keys (How many block). The rule that should be applied is presented in each trial at the top of the screen. In a second phase, two mixed blocks are presented (each consisting of 8 practice and 72 assessment trials). Participants are required to alternate between the aforementioned tasks (How many, What number). The rule that should be applied switch after every second trial. Trials are subject-paced and a variable response–stimulus interval of 0–300 ms is used. A 1000-Hz tone sounded for 300-ms on each error. Accuracy rates and mean RTs (on accurate responses) were obtained per condition: for pure blocks’ trials in the non-switch blocks and for switch and non-switch trials in the mixed blocks.

##### Verbal Fluency

Children were also given a verbal fluency task [136], which consisted of two assessment sessions: a semantic fluency and a phonological fluency session (see relevant assessments with children with DLD [38,39,68,102]). In the semantic fluency test, participants are asked to produce as many different words as they can for each of three semantic categories (animals, fruit, objects). In the phonological fluency assessment, children are asked to produce as many different words as they can that begin with each of the three Greek letters: X (Chi), Σ (Sigma), and A (Alpha). Participants are given a minute for each category/letter session. Sessions and categories were administered in the aforementioned order. The sum of relevant words produced in each session, excluding repetitions and variations of previously given words, provided measures of semantic and phonological fluency, accordingly, for each participant.

### 2.4. Procedure

Following informed parental consent, as well as children’s assent, participants were individually tested in the school setting in one preliminary and three main assessment sessions. Each session lasted approximately 30–40 min and took place on a different weekday, with the whole assessment procedure completed within a fortnight. In the first, preliminary session, children were given the Raven’s and the language tests to ensure appropriate inclusion of children in the DLD and the TD groups. In the sessions that followed, the cognitive tasks were administered in pseudorandom order, taking care that no similar tasks (e.g., the verbal WM or updating measures, or the two inhibition-related measures) were administered within the same session.

## 3. Results

### 3.1. Do Greek-Speaking School-Age Children with DLD Have Lower Scores Than TD Peers on Non-Verbal Intelligence?

A comparison of the standard non-verbal intelligence scores of the two groups (measured with the Raven’s Colored Progressive Matrices) revealed a significant difference and a large effect size (*p* < 0.001, *d* = 1.21; see Table 1). Specifically, children with DLD scored significantly lower than TD peers on the non-verbal intelligence test (mean difference = 12.86).

### 3.2. Are Greek-Speaking School-Age Children with DLD Outperformed by TD Peers in Tasks Tapping (a) WM Capacity; (b) Key EFs, That Is, Updating, the Resistance to Distractor Interference and Resistance to Proactive Interference Functions of Inhibition, and Task-Switching; as Well as (c) Verbal (Phonological and Semantic) Fluency (Executive Language Measures)?

Table 1 provides the descriptive statistics for the dependent variables of the study. A MANOVA was conducted to test the effect of group (TD vs. DLD) on participants’ WM capacity as tapped by the listening recall and the backward digit recall measures (*r* = 0.491, *p* < 0.001). Using Wilk’s lambda statistic, there was a significant effect of group on the measures, Λ = 0.63, *F*(2, 54) = 15.61, *p* < 0.001, η_p_^2^ = 0.366. Similarly, separate univariate ANOVAs revealed significant effects of group on the listening recall test, *F*(1, 55) = 28.1, *p* < 0.001, η_p_^2^ = 0.338, and the backward digit test, *F*(1, 55) = 12.59, *p* = 0.001, η_p_^2^ = 0.186. In both cases, mean performance was higher in the TD relative to the DLD group (see Table 1).

We proceeded with *t*-tests for independent samples to compare the two groups (TD vs. DLD) on the updating, the inhibition, and the verbal fluency measures (see Table 1 for statistics). In line with the patterns observed for WM capacity, comparisons between the two groups on updating gave a statistically significant difference and a moderate effect size regarding the number of targets recognized in the n-back task; there was no effect of group on participants’ mean RT for targets recognized. On the other hand, the results showed that the groups did not differ on either inhibition measure obtained (the resistance to distractor interference and the resistance to proactive interference measures). Yet, significant group differences were found in verbal fluency. In both the phonological and the semantic fluency subtests, the TD children demonstrated higher mean scores than DLD peers (mean differences were 12.19 and 6.86, respectively).

Finally, a two-way, mixed-design ANOVA was performed on participants’ RTs in the task-switching paradigm employed, with Group (TD vs. DLD) as the between-subjects factor and trial type (pure blocks’ trials vs. switch trials vs. non-switch trials) as the within-subjects factor. Mauchly’s test indicated that the assumption of sphericity had been violated for the main effects of shifting, *W* = 0.447, *p* < 0.001, ε = 0.64. Therefore, degrees of freedom were corrected using Greenhouse–Geisser estimates of sphericity. Both main effects were significant. Specifically, there was a significant main effect of trial type on mean RTs [*F*_shifting_(1.287, 72.098) = 113.86, *p* < 0.001, η_p_^2^ = 0.67]. There was also a significant main effect of group [*F*_group_(1, 56) = 12.24, *p* = 0.001, η_p_^2^ = 0.179] indicating that, overall, the TD group was faster than the DLD group.

**Figure 1:**
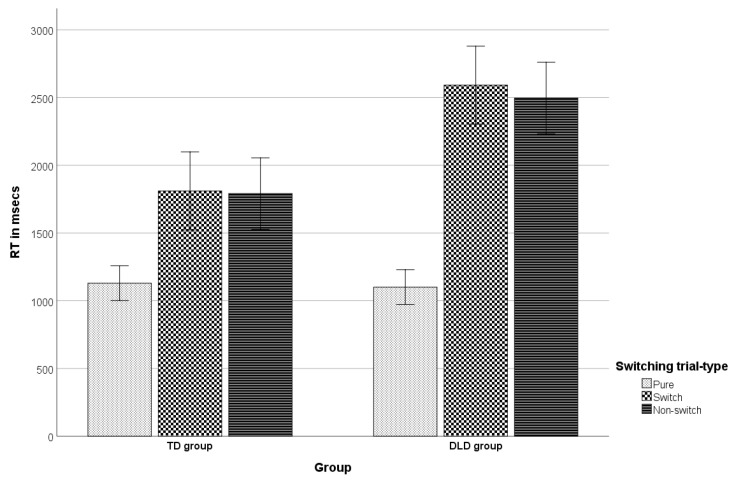
The group by shifting interaction (RT-estimated marginal means and error bars in msecs).

There was a significant interaction effect between trial type and group [*F*_shifting × group_ (1.287, 72.098) = 15.248, *p* < 0.001, η_p_^2^ = 0.214], indicating that group had different effects on children’s RTs depending on trial type. The interaction graph (see Figure 1) reveals that whereas mean RTs in the pure blocks’ trials (i.e., the blocks where participants did not switch at all) were similar for both groups (mean difference = 28.8 ms, *p* = 0.753), there were significant speed differences between groups in the switch and the non-switch (repeat) trials of the mixed, task-switching blocks. Specifically, the TD group was faster in both conditions (mean difference = 780.9 ms, *p* < 0.001 and mean difference = 705.7 ms, *p* < 0.001, respectively). On the other hand, within each group, children were significantly slower in the mixed blocks’ switch trials relative to the pure blocks’ trials: mean difference_DLD_ = 1490.9 ms, *p* < 0.001 and mean difference_TD_ = 681.3 ms, *p* < 0.001. A significant difference was not observed between the mean RTs in the switch and the non-switch trials in either group: mean difference_DLD_ = 95 ms, *p* = 0.205 and mean difference_TD_ = 19.9 ms, *p* = 0.625. However, in both groups, children were much slower in the non-switch trials of the mixed blocks relative to the pure blocks’ trials: mean difference_DLD_ = 1395.9 ms, *p* < 0.001 and mean difference_TD_ = 661.4 ms, *p* < 0.001. This indicates a significant mixing cost in each group [137,138,139], which, based on a *t*-test for independent samples, actually proved significantly smaller in magnitude in the TD group (661.43 ms) relative to the DLD group (1395.87 ms), *t*(56) = −4.03, *p* < 0.001, *d* = −1.06. The latter finding, however, could only be discussed with caution, due to the lack of a significant switching cost (the alternative cost of multitasking) in either group tested with the specific paradigm. Given the inability to cleanly separate the two cost types in the present study, we proceeded to a comparison of the general performance difference between pure and mixed blocks. It is noted that such comparisons have also been approached as indicative of mixing costs, reflecting general monitoring capacity and top-down processing [140,141] (see discussion in Philipp et al. [142]). The analysis showed that children were overall much slower in the mixed blocks’ relative to the pure blocks’ trials (mean difference_DLD_ = 1443.39 ms, *p* < 0.001 and mean difference_TD_ = 671.37 ms, *p* < 0.001), with the magnitude of the difference found significantly greater in the TD group (661.43 ms) relative to the DLD group (1395.87 ms), *t*(56) = −4.18, *p* < 0.001, *d* = −1.1.

### 3.3. Are Any Group Differences in WM Capacity, EFs, and Verbal Fluency Independent of Non-Verbal Intelligence?

Aiming to examine whether the observed group differences are independent of non-verbal intelligence, one-way analyses of covariance (ANCOVA), with the standard non-verbal intelligence score entered as a covariate, were additionally performed on all measures. We are not reporting these analyses for two reasons: (a) the homogeneity of regression slopes ANCOVA assumption was not met in several cases (specifically, in the case of the two working-memory measures, in the switch and the non-switch trials of the mixed blocks of the task-switching paradigm, and in phonological fluency), and (b) the results of the ANCOVAs did not differentiate any patterns observed in the *t*-tests, the MANOVA, and the mixed ANOVA reported above.

Thus, concerning the significant differences observed between groups in cognitive measures (see the previous section), we can conclude the following. Relying on the ANCOVAs that could be conducted, it seems that the group differences observed in the updating (targets recognized) and the semantic fluency measures can be approached as independent of non-verbal intelligence. On the other hand, in the cases that the ANCOVA results mentioned above (i.e., lack of patterns’ differentiation) cannot be reliably discussed, since the homogeneity of regression slope assumption was not met, we can only rely on the correlations between non-verbal intelligence and each cognitive measure to discuss possible dependence of group differences on non-verbal intelligence. Specifically, the latter showed a significant correlation with (a) the digit backwards measure in the DLD group only (*r* = 0.44, *p* = 0.016), (b) mean RT in the non-switch trials of the task-switching paradigm in the TD group only (*r* = −0.38, *p* = 0.044), and (c) the related mixing cost measure (pure vs. mixed blocks’ RTs) in the TD group only (*r* = −0.40, *p* = 0.031). There were no significant correlations between the non-verbal intelligence score and the listening recall, the phonological fluency, or speed (RT) of response in the switch trials of the relevant paradigm in either group tested (*p* > 0.05). Overall, our findings seem to suggest a limited possible interplay between non-verbal intelligence and differentiation of WM andEFs in children with DLD relative to TD peers. Effects seem limited to the backward digit recall (WM) measure and certain task-switching indices (non-switch trials’ RT and mixing cost), yet related to DLD only in the case of the specific WM measure.

## 4. Discussion

The aim of the present study was to investigate WM capacity, key EFs, and verbal fluency in relation to non-verbal intelligence in Greek-speaking school-age children with DLD in comparison to TD peers. To our knowledge, this is the first study that has attempted the combined assessment and comprehensive investigation of the aforementioned capacities in the specific population. Our first research question regarded possible differences between children with DLD and TD peers in non-verbal intelligence, despite both groups being of average intelligence (standard score of 85 or above in the Raven’s Educational CPM/CVS). In line with the hypothesis stated, children with DLD scored lower on the non-verbal intelligence test relative to TD peers. This is consistent with the meta-analysis of Gallinat and Spaulding [12], in which children with DLD scoredon average 10 points below TD peers [56]. It seems that lower—even if average—non-verbal intelligence may be a part of the clinical profile of children with DLD. Future research is needed to gain a better understanding of the role of non-verbal intelligence in DLD and resolve the relevant, ongoing debate regarding DLD diagnosis.

Our second research question regarded the differentiation of children with DLD and TD peers in measures of WM capacity, key EFs, and verbal fluency. We opted for a general hypothesis, given inconclusive relevant findings as well as scarce evidence with the specific population (i.e., Greek-speaking school-age children with DLD). We expected differences in favor of the TD children in the measures obtained that would be independent of non-verbal intelligence (see relevant evidence in Kuusisto et al. [56]). Our hypothesis was confirmed in several cases, though there were some exceptions. That is, we did observe differences in favour of the TD group in several cognitive measures: specifically, in both WM capacity measures and the executive WM measure (updating, as tapped by the number of targets recognized), in mean RTs in the switch and the non-switch (mixed blocks’) trials of the task-switching paradigm, and the magnitude of the mixing cost in the same task (mixed vs. pure block’s RTs; though we did not observe a switching cost, see discussion below), as well as in both verbal fluency (phonological and semantic) measures obtained. There were no group differences though, in the inhibition measures (tapping resistance to distractor and resistance to proactive interference) or the speed at which targets were recognized in the updating task. Moreover, our findings seem to suggest limited possible interplay between non-verbal intelligence and differentiation of WM and EFs in children with DLD and TD peers. Relying on the ANCOVAs that were conducted, it seems that the aforementioned differences in updating (targets recognized) and semantic fluency persisted even when adjusting for any non-verbal intelligence effects. On the other hand, where ANCOVA results could not be reliably discussed (see Results), the examination of the correlation between non-verbal intelligence and each cognitive measure suggested a possible role of non-verbal intelligence in the differentiation of groups only in the cases of backward digit recall and certain task-switching indices (non-switch trials RT and mixing cost). However, the effect seemed related to DLD in the case of the specific WM measure only (only the latter correlated with non-verbal intelligence among children with DLD). The findings of this preliminary investigation with Greek-speaking school-age children—an under-examined population so far—are further discussed below in relation to the predominant results’ patterns in the literature and relevant theoretical suggestions, also directing the reader to future research.

Specifically, children with DLD were outperformed by TD peers in (a) WM capacity (complex span tasks performance), in line with studies involving children that speak other languages (mostly English [2,68,69]); and (b) executive WM, that is, updating (as tapped by targets recognized in the 2-back task), for which evidence remains scarce and inconclusive in relation to DLD (e.g., school-age children performed at chance in a 2-back task in the Im-Bolter et al. [76], e.g., null findings were obtained in the Lukács et al. study [39]). There were no group differences, though, in the speed measure regarding recognition of targets in the updating task. Though the latter was not our primary updating index, it helped to clarify that participants with DLD were outperformed on accuracy rather than response speed for targets correctly identified. One limitation of the present study is that we did not also employ visuospatial WM tasks (that is, complex span or updating tasks), given assessment duration limitations. Yet, results’ patterns seemed aligned for both the listening recall measure (setting linguistic processing demands) as well as the backward digit recall measure and the 2-back task with digits used to measure updating (which set minimum demands for manipulation or processing of linguistic information). It should also be noted that recent studies have pointed to domain-general WM capacity constraints when using verbal and non-verbal complex span tasks to assess children with DLD [40,68]. Moreover, the nature of the stimuli presented (verbal vs. non-verbal) in n-back tasks did not differentiate the patterns of findings reported by Lukács et al. [39]; it is noted, however, that null findings were obtained in the latter study, in contrast to the present study. Future studies can further explore whether WM difficulties in DLD are related to WM capacity (executive control in WM as tapped by complex-span tasks) and/or the executive WM updating function. Such exploration is of value as it can inform the theoretical as well as clinical characterization of the WM—linguistic limitations association in children with DLD (see [2] for a discussion). Future research can also guide the refinement of diagnostic tools and treatment methods to enhance WM functioning in this population. As Archibald [143] recently proposed, every child with DLD may not have WM deficits, but when he/she does face severe relevant deficits, these might constitute an underlying cause of linguistic impairment (e.g., lexical and grammatical difficulties in [144]). On this basis, future research, as well as clinical practice should consider the employment of cognitive measures as well, in accurately interpreting the linguistic performance of children with DLD.

On the other hand, the two groups of children did not differ in the two inhibition-related measures employed in the present study, despite earlier suggestions for WM deficits in DLD co-occuring or even resulting from inefficient inhibition [145]; although, as Marton et al. [146] noted, the direction of causality remains unclear. Specifically, our findings regarding resistance to distractor interference are aligned with those of certain studies [78], but not others, showing significant group differences in favour of TD children [83,146]. Based on the findings of the latter, it has been suggested that deficient interference resolution hinders the ability of children with DLD to resolve conflict among semantic representations that are activated during language processing [147]. Such a suggestion, however, is not supported by the present study; whereas our conflicting results regarding WM capacity (significant group differences in favour of the TD group) and inhibition (null findings) are not supportive of suggestions that the difficulties of children with DLD in verbal WM tasks reflect poor inhibition (resistance to distractor and proactive interference) [82,84]. Future studies including children of different ages can show whether lack of group differences in the inhibition measures employed in the present study is related to our participants’ age (8.9 years; 3rd graders) and prioritization of interference resolution capacities in later phases of development. There is evidence to suggest, for example, that interference resolution develops later than response inhibition (not measured in the present study though) in TD children [148] and that sustained attention and inhibition remain immature until 8 years of age [82,149]. Given the importance of the inhibition-related functions for language processing and learning more generally [82,150], age-related changes in inhibition merits future exploration in children with DLD as well.

With regard to task switching, although the two groups did not differ in the baseline condition (pure blocks), children with DLD had slower mean RTs than TD peers in both complex blocks’ conditions (switch and non-switch trials). Therefore, their performance seemed more greatly influenced by the complexity and demands of this mixed block, necessitating strategic engagement to deal with maintenance of multiple task sets. Moreover, both groups responded significantly slower in the trials of the complex blocks relative to the pure blocks’ trials. Notably, the magnitude of this mixing cost was significantly larger in the DLD relative to the TD group, indicating difficulties in time-sensitive shifting of mental sets, as well as worse general monitoring capacity and top-down processing in children with DLD [139,140]. However, as noted above, the present finding is interpreted with caution, and merely as a tendency that merits further investigation, given the lack of significant switch costs in the task [151]. Whether the latter resulted from the significant demands placed on very young children by the specific paradigm could be examined in future studies involving children of different ages in different switching tasks, besides the present. Further investigation is, in any case, necessitated to more cleanly separate mixing from switching costs in the study of the specific EF as a function of DLD.

Finally, TD children outperformed peers with DLD in both the phonological and semantic fluency measures obtained, with differences between the groups not proving dependent on non-verbal intelligence. As noted, the ANCOVA conducted for the semantic fluency measure with non-verbal intelligence as a covariate, did not change the results’ pattern; whereas the phonological fluency measure was not related to non-verbal intelligence in either group tested. In children with DLD, poorer phonological fluency has been attributed to impaired explicit (vs implicit) access to phonological representations (Deficient Phonological Access Hypothesis) [152], whereas poorer semantic fluency has been related to a general slowing of retrieval processes in contrast to intact lexical-semantic representations (Retrieval Slowing Model) [153]. However, existing studies have mostly relied on overall verbal fluency measures [39,68]. Moreover, findings remain limited to languages other than English, although language characteristics can affect performance in tasks tapping on lexical access and vocabulary development [63]. In line with our results on semantic fluency (yet, contrast to findings in studies with English-speaking children [100,101]), a recently published study [102] found poorer semantic fluency in Greek-speaking school-age children with DLD relative to TD peers. However, researchers did not additionally obtain a phonological fluency measure.

Future research could also consider including different DLD subtypes, that is, groups identified on the basis of expressive difficulties (like the present sample) as well as receptive-expressive groups, to help disentangle the language-cognitive interplay in DLD. For example, in one of the few relevant studies, Rodríguez et al. [40] demonstrated problems with verbal fluency and WM (verbal and spatial) in the expressive DLD group, yet, poorer neuropsychological performance more generally, in the receptive-expressive DLD group.

## 5. Conclusions

The present study aimed at investigating WM capacity, key EFs, and verbal fluency indices in relation to non-verbal intelligence in children with DLD, relative to TD peers; following the limited in number comprehensive investigations of cognitive capacities in this population, and attempting a relevant exploration with Greek-speaking, school-age children with DLD, for the first time. In line with increasing relevant findings (see meta-analysis of Gallinat and Spaulding [12], Kuusisto et al. [56]) and our first hypothesis, children with DLD scored lower than TD peers on non-verbal intelligence. Moreover, we expected differences in favor of the TD children on the WM capacity, EF, and verbal fluency measures, that would be independent of non-verbal intelligence. Our second hypothesis was partially confirmed. Our findings suggest lack of group differences in the inhibition-related functions studied (resistance to distractor interference and resistance to proactive interference). However, we found differences in favour of the TD group in the WM capacity (both complex span tasks), updating (targets recognized in the 2-back task), monitoring (mixing cost in the switching paradigm), and verbal fluency (phonological and semantic) measures, which did not seem dependent on non-verbal intelligence. The only group difference that showed possible dependence on non-verbal intelligence as a function of DLD, was that on the backward digit recall (WM) measure.

Future studies should further investigate the generality of the differences observed between groups in high-order cognitive capacities, employing verbal as well as non-verbal tasks [68], and extending assessment to other capacities, such as planning. Moreover, longitudinal research can investigate the developmental trajectories of higher-order cognitive processes and language skills in children with DLD [56], and help disentangle whether language and cognitive development is underlined by common mechanisms (e.g., WM and attentional processes) in children with DLD [12,154]. Finally, cross-linguistic research can explore universal, as well as possibly, language-specific features, in DLD profiles [155]. Such investigations can further inform clinical practice for children with DLD, contributing to more accurate mappings of WM, EFs, and verbal fluency capacities to language profiles, thus, enhancing screening procedures and the effectiveness of intervention programmes [3].

## Figures and Tables

**Table 1:** Descriptive statistics and *t*-test results.

	TD		DLD		
	Mean (SD)		Mean (SD)		*t*-Tests and Effect Sizes
Non-verbal intelligence (Std. Sc.)	132.86	(11.7)	120.0	(9.5)	*t*(56) = 4.6, *p* < 0.001, *d* = 1.21
WM capacity					
Listening recall	14.9	(4.6)	9.32	(3.2)	See MANOVA results in Section 3.2
Backward digit recall	15.07	(3.9)	11.21	(4.2)	
Updating					
Targets recognized (sum)	12.28	(3.4)	10.16	(3.6)	*t*(52) = 2.21, *p* = 0.032, *d* = 0.60
Targets recognized (RT)	939.21	(342.8)	865.04	(261.2)	*t*(52) = 0.88, *p* = 0.381, *d* = 0.24
Inhibition					
Resistance to distractor interference	99.59	(221.0)	169.69	(200.3)	*t*(56) = −1.266, *p* = 0.211, *d* = −0.33
Resistance to proactive interference	2.67	(1.8)	3.34	(1.9)	*t*(56) = −1.38, *p* = 0.172, *d* = −0.36
Task switching					
Pure blocks’ trials (RT)	1129.41	(355.8)	1100.66	(336.5)	See mixed ANOVA results in Section 3.2
Switch trials (RT)	1810.72	(675.7)	2591.57	(858.8)	
Non-switch trials (RT)	1790.85	(669.4)	2496.53	(746.9)	
Verbal fluency					
Phonological	27.55	(9.0)	15.36	(7.2)	*t*(55) = 5.638, *p* < 0.001, *d* = 1.49
Semantic	40.69	(11.7)	33.83	(9.1)	*t*(56) = 2.49, *p* = 0.016, *d* = 0.65

## Data Availability

Data is available on request.
